# An Endostatin-lentivirus (ES-LV)-EPC gene therapy agent for suppression of neovascularization in oxygen-induced retinopathy rat model

**DOI:** 10.1186/s12860-020-00301-1

**Published:** 2020-07-29

**Authors:** Jing Ai, Jian Ma, Zhi-Qing Chen, Jun-Hui Sun, Ke Yao

**Affiliations:** 1 Eye Center, The Second Affiliated Hospital, Zhejiang University School of Medicine, Hangzhou, 310009 Zhejiang Province China; 2 Hepatobiliary and Pancreatic Interventional Treatment Center, Division of Hepatobiliary and Pancreatic Surgery, Key Laboratory of Combined Multi-organ Transplantation, Ministry of Public Health, The First Affiliated Hospital, Zhejiang University School of Medicine, Hangzhou, 310003 Zhejiang Province China

**Keywords:** Retinal neovascularization, Endothelial progenitor cells, Gene therapy, Endostatin

## Abstract

**Background:**

Transplantation of gene transfected endothelial progenitor cells (EPCs) has provided novel methods for tumor neovascularization therapy but not for ocular disease therapy. This study aimed to investigate the efficacy of endostatin transfected EPCs in retinal neovascularization therapy.

**Results:**

Quantitative reverse transcription-polymerase chain reaction (qRT-PCR) showed the high expression of endostatin in endostatin-lentivirus-EPCs. The neovascularization leakage area and the number of preretinal neovascular cell nuclei were significantly decreased in the endostatin-lentivirus and endostatin-lentivirus-EPC groups, and the effects of these two treatments on inhibiting retinal neovascularization were almost the same. These two groups also showed the greater retinal distribution of endostatin. Intravitreal injections of endostatin-lentivirus-EPCs inhibited retinal neovascularization, vascular endothelial growth factor (VEGF) and CD31 expression, and increased endostatin expression in vivo. Endostatin-lentivirus-EPCs targeted and prevented pathologic retinal neovascularization.

**Conclusions:**

Gene-combined EPCs represent a potential new therapeutic agent for the treatment of neovascular eye diseases.

## Background

Retinal neovascularization is a severe complication in most types of retinopathy, such as proliferative diabetic retinopathy, retinopathy of prematurity, and age-related macular degeneration [1]. It is the first leading cause of vision impairment and irreversible blindness today. Although conventional therapies for retinal neovascularization include surgical vitrectomy, laser photocoagulation, photodynamic therapy, and intravitreal injection of anti-VEGF factors, which are widely available. The prognosis for retinal neovascularization remains extremely poor, and retinal neovascularization recurrence rates remain high; only a few patients achieve good vision without recurrences [1–3]. This outcome can be traced back to the finding that neovascular tissue has a distinct ability to secrete growth factors such as vascular endothelial growth factor (VEGF) and to infiltrate the blood-retinal barrier or to disrupt the extracellular matrix [4]. Thus, it eventually leads to retinal neovascularization recurrence following multiple initial treatments [4, 5]. Therefore, novel therapeutic methods for patients with retinal neovascularization are required for improving therapy outcomes.

Previous studies indicated that anti-VEGF agents can inhibit ocular neovascularization through intravitreal injections [6–9]. Bevacizumab and ranibizumab have been reported to decrease optic disc edema or to have an anti-inflammatory and anti-neovascular effect on neovascular age-related macular degeneration [6–8]. However, expensive charges, frequent office visits, and multiple injections are added to the burden of patients with this condition. The injections are also associated with a low risk of an increase in intraocular pressure, and the incidence of vitreous hemorrhage, uveitis, vascular occlusion, or the retinal detachment is elevated [9–11]. Worse, neovascularization may reappear when the therapy is over because the effect of a single injection of anti-VEGF agents is temporary [12].

Continuous suppression of neovascularization may be more efficacious than monthly injections of anti-VEGF [13]. Moreover, there is a big difference in patients’ responses to treatment. Approximately 10% of patients do not respond to anti-VEGF therapy in spite of receiving monthly intravitreal injection therapy for 2 years [8, 12]. Therefore, based on the advantages of gene therapy, the local and sustained delivery of anti-angiogenic molecules is feasible, and it has been confirmed in animal models that neovascularization can be efficiently suppressed by gene therapy [13–15]. In the present study, the endostatin gene was selected due to its profound effects on angiogenesis. Firstly, endostatin is an angiogenesis inhibitor that inhibits endothelial cell (EC) proliferation, migration or invasion, blocks the formation of new blood vessels, and decreases retinal VEGF expression [16]; secondly, endostatin can be secreted by many cells but has no effect on the blood vessels around normal tissue [17]. Endostatin is non-toxic and has no drug resistance [16, 17]. Although the amount of endogenous endostatin increases in proliferative diabetic retinopathy, this increase is not enough to inhibit retinal neovascularization [3]. Therefore, it is important for the amount of endostatin expression to be increased in vivo, and gene therapy enables this.

Endothelial progenitor cells (EPCs), which exist primarily in bone marrow, can migrate from blood circulation to ischemic or neovascular sites, have a high proliferative rate, and differentiate into ECs [18]. Under normal physiological conditions, healthy EPCs can be applied to repair ischemic vascular damage [19, 20]. However, under the pathological condition of neovascularization, the amount of EPCs increases, but their biological function is not improved; hence, the EPCs cannot repair the vascular endothelium. The mobilized EPCs may promote the formation of new blood vessels [21–23], so the transplantation of healthy EPCs is essential [24].

EPCs represent an effective delivery vehicle for gene therapy against neovascular formation by virtue of their mobilization [24, 25]. The potential for EPCs to serve as cellular vehicles for molecular therapy against neovascularization depends on efficient and specific gene transfer and the ability to stably deliver therapeutic loads through the blood to the intended target [25]. It has been reported that the angiogenic gene (VEGF) transfected EPCs migrate to and increase the blood supply to sites of vascular injury [26]. Therefore, it can be inferred that the transplantation of endostatin-lentivirus -EPCs to retinal tissue may not only provide a sufficient number of healthy EPCs to the target area but also may inhibit the VEGF expression level and the migration of ECs via endostatin expression of endostatin-lentivirus-EPCs. The high VEGF level in the neovascular regions could be fundamentally solved through this strategy. Thus, the development of retinal neovascularization could be improved or reversed.

The aims of the present study were as follows: to establish a novel therapeutic modality using EPCs as vehicles to target neovascular vessels and to secrete endostatin; to investigate whether intravitreal injection of endostatin-lentivirus-EPC is effective for treating retinal angiogenesis in an animal model of oxygen-induced retinopathy (OIR) and to test our hypothesis; and to evaluate the feasibility of this technique in clinical therapy for retinal neovascularization.

## Results

### Fluorescent images of EPCs

**Fig. 1:**
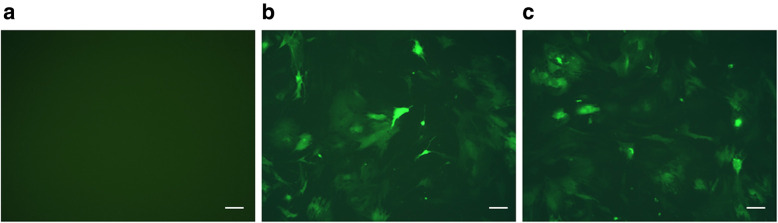
EPCs photographed by fluorescence microscopy (magnification × 100). **a** Non-transduced EPCs. **b** EPCs transduced with lentivirus-GFP. **c** EPCs transduced with endostatin-lentivirus-GFP. GFP: green fluorescent protein. Scale bar = 50 μm

There was no difference between EPCs transduced with endostatin-lentivirus-GFP and EPCs transduced with lentivirus-GFP. No fluorescence was observed in non-transduced EPCs (Fig. 1).

### Endostatin expression in EPCs

**Fig. 2:**
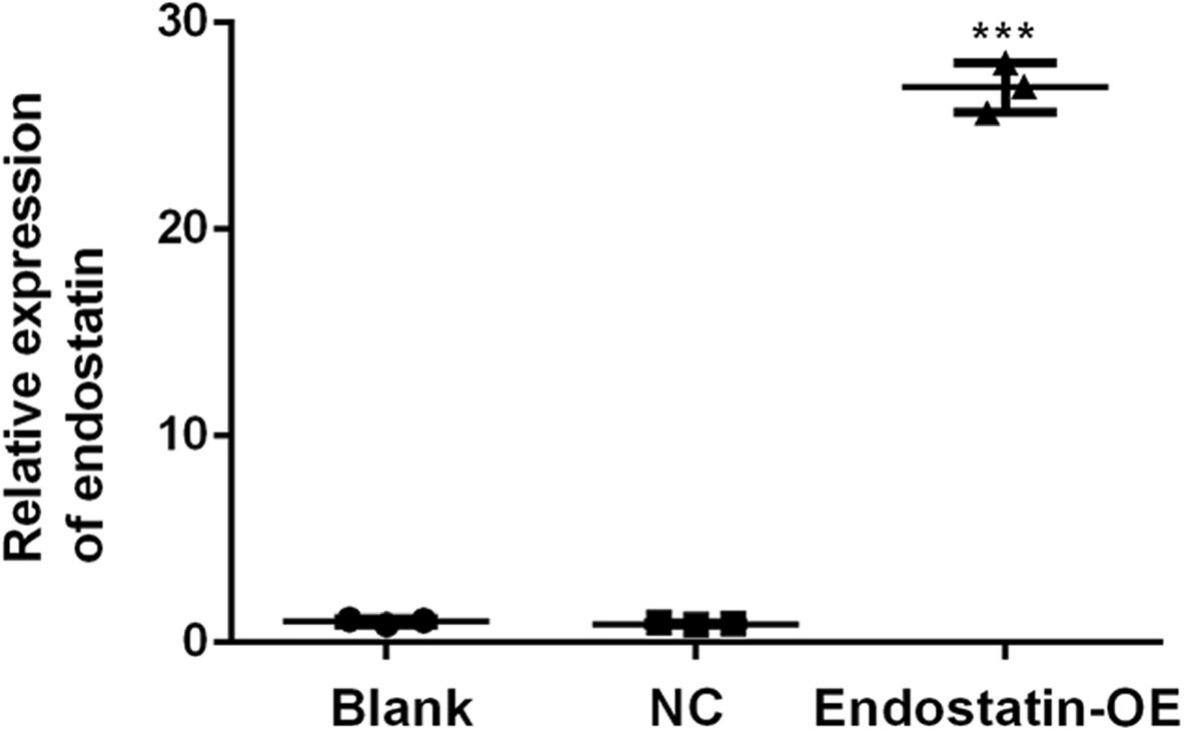
Relative mRNA level of endostatin in EPCs. ***P < 0.001 as compared with the NC. Blank: blank control group (non-transduced EPCs); NC: negative control group (EPCs transduced with lentivirus-GFP); Endostatin-OE: endostatin-overexpression group (EPCs transduced with endostatin-lentivirus-GFP); GFP: green fluorescent protein

The total RNA was extracted from EPCs using TRIzol® reagent (Invitrogen; Carlsbad, CA, USA), according to the manufacturer’s instructions. The straps were cleared at 28S rRNA and 18S rRNA on agarose gels. The value of optical density (260: 280 nm) was 1.9–2.2. EPCs transduced with a lentiviral vector encoding endostatin-GFP resulted in endostatin overexpression. The endostatin mRNA expression in the endostatin overexpression group increased significantly (*P* < 0.001), as compared with that in the NC group. However, there was no difference in mRNA expression between the blank control and NC groups (Fig. 2).

### Inhibition effects of endostatin-lentivirus on retinal neovascularization

#### Fundus fluorescein angiography results

**Fig. 3:**
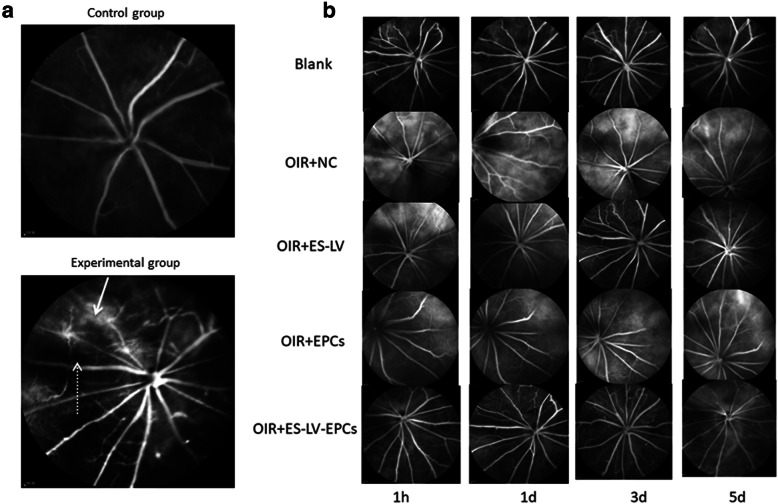
Fundus fluorescein angiography representative photos. **a** A capillary non-perfusion zone and neovascularization leakage were observed in the experimental group but not in the control group. Fluorescein leakage in the neovascular area (indicated by a white arrow); capillary non-perfusion area (indicated by a dashed white arrow). **b** After intravitreal injection, representative photos of one eye were observed by fundus fluorescein angiography at different time points (1 h, 1 d, 3 d, and 5 d). Blank: blank control group (age-matched rats kept in normoxia with non-intravitreal injection); OIR + NC (negative control group): oxygen-induced retinopathy (OIR) rats with empty-lentivirus intravitreal injection; OIR + ES-LV: OIR rats with endostatin-lentivirus intravitreal injection; OIR + EPCs: OIR rats with EPCs intravitreal injection; OIR + ES-LV-EPCs: OIR rats with endostatin-lentivirus-EPCs intravitreal injection

A capillary non-perfusion zone and neovascularization leakage were observed in the experimental group but not in the control group (Fig. 3a).

**Fig. 4:**
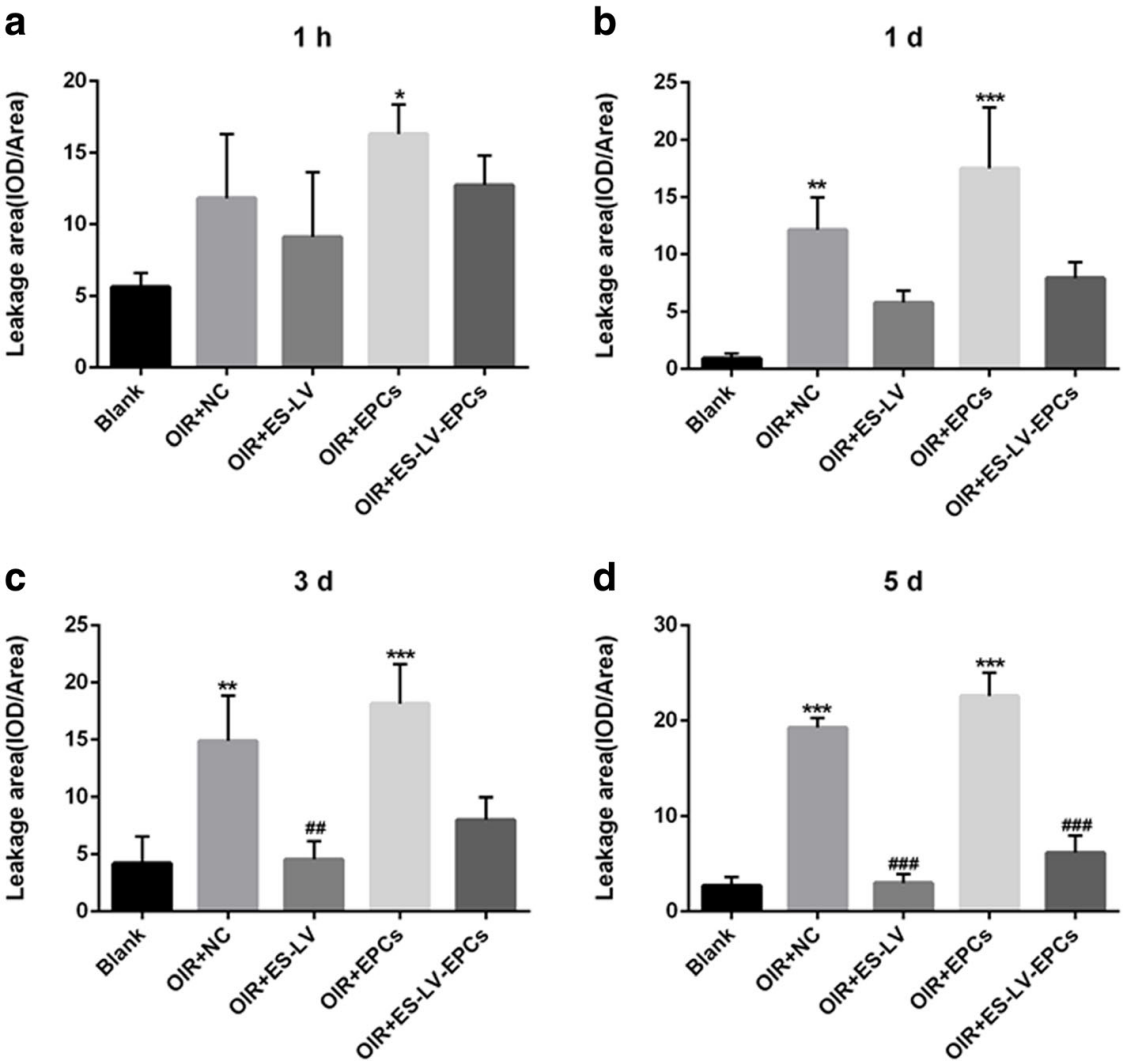
Neovascularization leakage areas analysis (IOD/Area, comparison in 5 groups). **a** Neovascularization leakage areas analysis at 1 h. **b** Neovascularization leakage areas analysis at 1d. **c** Neovascularization leakage areas analysis at 3d. **d** Neovascularization leakage areas analysis at 5d. ^*^P < 0.05, ^**^P < 0.01 as compared with Blank; ^##^*P* < 0.01, ^###^*P* < 0.001 as compared with OIR + NC. IOD: integral optical density. Blank: blank control group (age-matched rats kept in constant normoxia with non-intravitreal injection); OIR + NC (negative control group): oxygen-induced retinopathy (OIR) rats with empty-lentivirus intravitreal injection; OIR + ES-LV: OIR rats with endostatin-lentivirus intravitreal injection; OIR + EPCs: OIR rats with EPCs intravitreal injection; OIR + ES-LV-EPCs: OIR rats with endostatin-lentivirus-EPCs intravitreal injection

Compared with the blank control group (age-matched rats kept in normoxia with non-intravitreal injection), the amount of retinal neovascularization leakage significantly increased in the OIR + NC group (empty-lentivirus injection) on days 1, 3 (*P* < 0.01), and 5 (*P* < 0.001) but did not increase in the OIR + endostatin-lentivirus group (endostatin-lentivirus injection) at the above three time points. Compared with the OIR + NC group, the amount of retinal neovascularization leakage significantly reduced on days 3 (*P* < 0.01) and 5 (*P* < 0.001; Figs. 3b and 4). Therefore, it can be inferred that retinal neovascularization leakage was inhibited in the OIR+ endostatin-lentivirus group and that endostatin-lentivirus played a role in inhibiting retinal neovascularization leakage.

#### Hematoxylin-eosin (HE) results

**Fig. 5:**
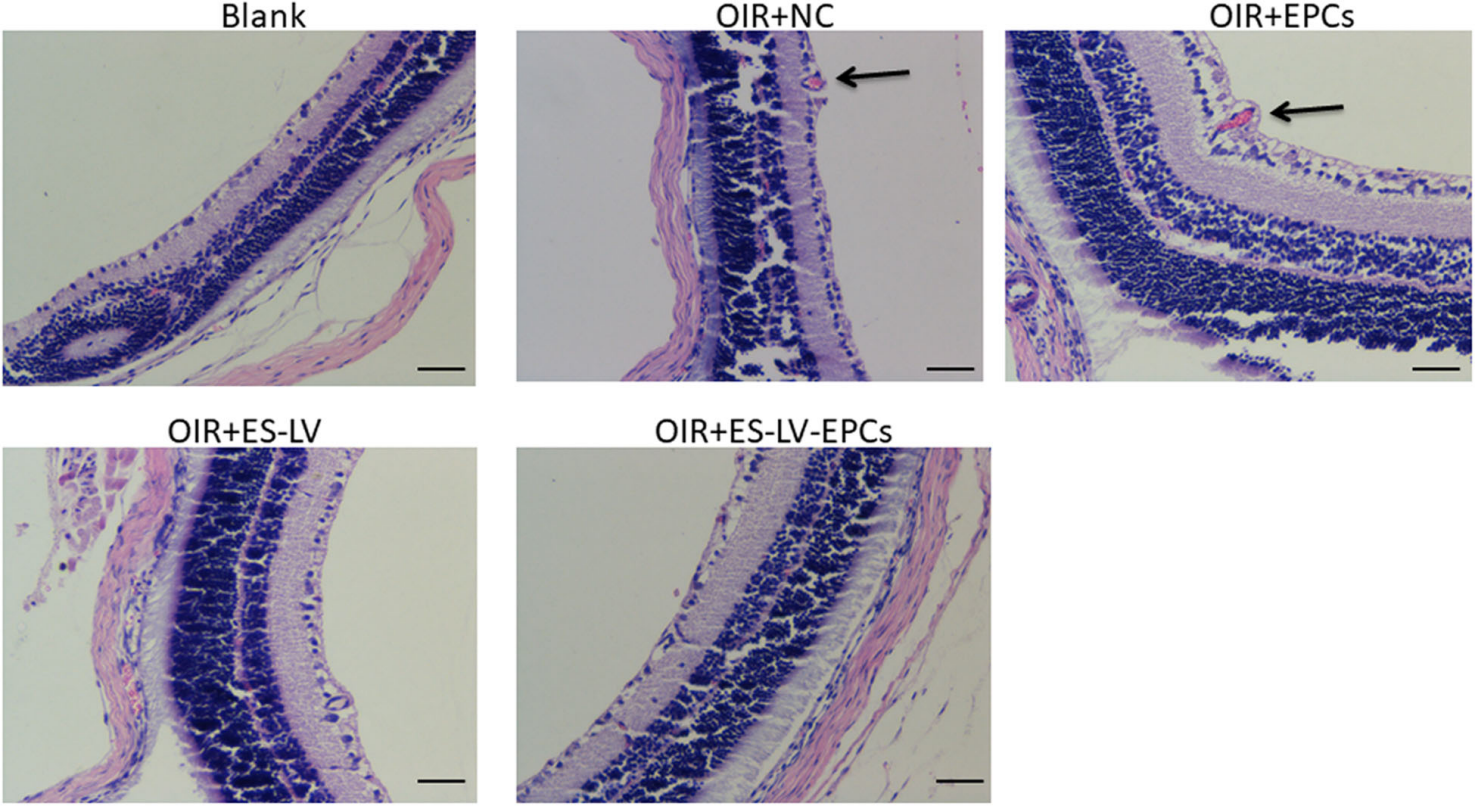
Representative images of retinal cross-sections stained with hematoxilin/eosin (magnification× 200). Black arrows indicate the preretinal neovascular cells on the vitreous side of the internal limiting membrane. Blank: blank control group (age-matched rats kept in constant normoxia with non-intravitreal injection); OIR + NC (negative control group): oxygen-induced retinopathy (OIR) rats with empty-lentivirus intravitreal injection; OIR + ES-LV: OIR rats with endostatin-lentivirus intravitreal injection; OIR + EPCs: OIR rats with EPCs intravitreal injection; OIR + ES-LV-EPCs: OIR rats with endostatin-lentivirus-EPCs intravitreal injection. Scale bar =25 μm

**Fig. 6:**
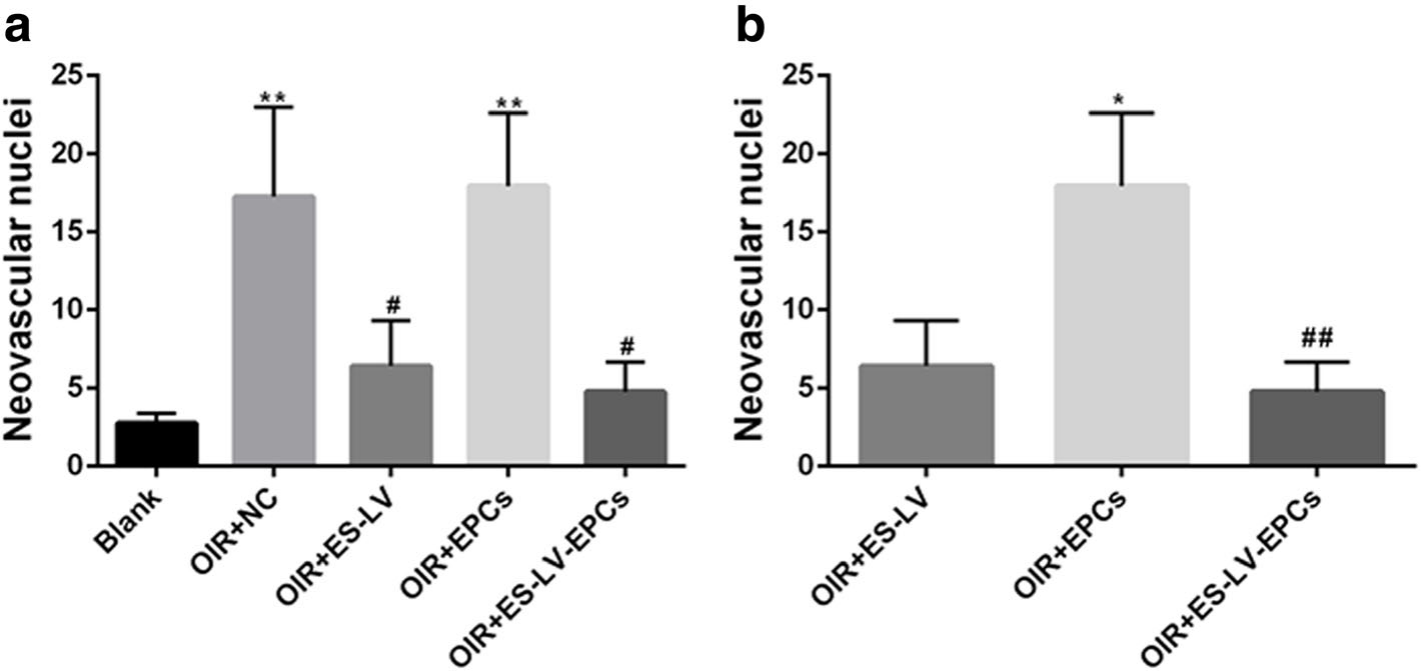
Assessment of preretinal neovascular cell nuclei in the vascular endothelium on the vitreous side. a^**^P < 0.01 as compared with Blank; ^#^*P* < 0.05 as compared with OIR + NC. b^*^P < 0.05 as compared with OIR + ES-LV; ^##^*P* < 0.01 as compared with OIR + EPCs. Blank: blank control group (age-matched rats kept in normoxia with non-intravitreal injection); OIR + NC (negative control group): oxygen-induced retinopathy (OIR) rats with empty-lentivirus intravitreal injection; OIR + ES-LV: OIR rats with endostatin-lentivirus intravitreal injection; OIR + EPCs: OIR rats with EPCs intravitreal injection; OIR + ES-LV-EPCs: OIR rats with endostatin-lentivirus-EPCs intravitreal injection

In comparison with the blank control group, the nuclei in the vascular endothelium increased significantly in the OIR + NC group (*P* < 0.01) but not in the OIR+ endostatin-lentivirus group. Compared with the OIR + NC group, the nuclei in the vascular endothelium decreased significantly in the OIR+ endostatin-lentivirus group (*P* < 0.05; Figs. 5 and 6a), so it can be inferred that retinal neovascularization was inhibited in the OIR+ endostatin-lentivirus group. The HE results agreed with the fundus fluorescein angiography results that endostatin-lentivirus helped in inhibiting retinal neovascularization leakage.

### Effects of simple EPCs on retinal neovascularization

#### Fundus fluorescein angiography results

The amount of retinal neovascularization leakage significantly increased at each time point (*P* < 0.05) in the OIR + EPC group (EPCs injected), as compared with the blank control group; but there were no significant differences at different time points (hour 1 and days 1, 3, and 5), as compared with the OIR + NC group (Figs. 3b and 4). Therefore, it can be inferred that simple EPCs have no effect on inhibiting retinal neovascularization leakage or promoting neovascularization.

#### HE results

Compared with the blank control group, the nuclei in the vascular endothelium increased significantly in the OIR + EPC group (*P* < 0.01); however, there was no significant difference between the NC and OIR + EPC groups (Figs. 5 and 6a), so it can be inferred that simple EPCs have no effect on inhibiting retinal neovascularization leakage. In addition, simple EPCs could not promote neovascularization.

### Inhibition effects of endostatin-lentivirus-EPCs on retinal neovascularization

#### Fundus fluorescein angiography results

In comparison with the blank control group, the amount of retinal neovascularization leakage significantly increased in the OIR + NC group (empty-lentivirus injection) on days 1, 3 (*P* < 0.01), and 5 (*P* < 0.001) but did not increase significantly in the OIR + endostatin- lentivirus-EPC group (endostatin-lentivirus-EPCs injection) at each time point (hour 1 and days 1, 3, and 5); as compared with the OIR + NC group, the amount of retinal neovascularization leakage significantly decreased on day 5 (*P* < 0.001; Figs. 3b and 4). Hence, it can be inferred that retinal neovascularization leakage was inhibited in the OIR+ endostatin-lentivirus-EPC group. Although endostatin-lentivirus-EPCs play a role in inhibiting retinal neovascularization leakage, inhibition occurs a little later than it does for endostatin-lentivirus.

#### HE results

Compared with the blank control group, the nuclei in the vascular endothelium increased significantly in the OIR + NC group (*P* < 0.01) but did not increase in the OIR + endostatin-lentivirus-EPC group. The nuclei in the vascular endothelium decreased significantly in the OIR+ endostatin-lentivirus-EPC group (*P* < 0.05; Figs. 5 and 6a) in comparison with the OIR + NC group, so it can be inferred that retinal neovascularization was inhibited in the OIR + endostatin-lentivirus-EPC group. The HE and fundus fluorescein angiography results were in agreement.

### Comparison between endostatin-lentivirus and endostatin- lentivirus-EPCs in inhibition effects on retinal neovascularization

#### Fundus fluorescein angiography results

**Fig. 7:**
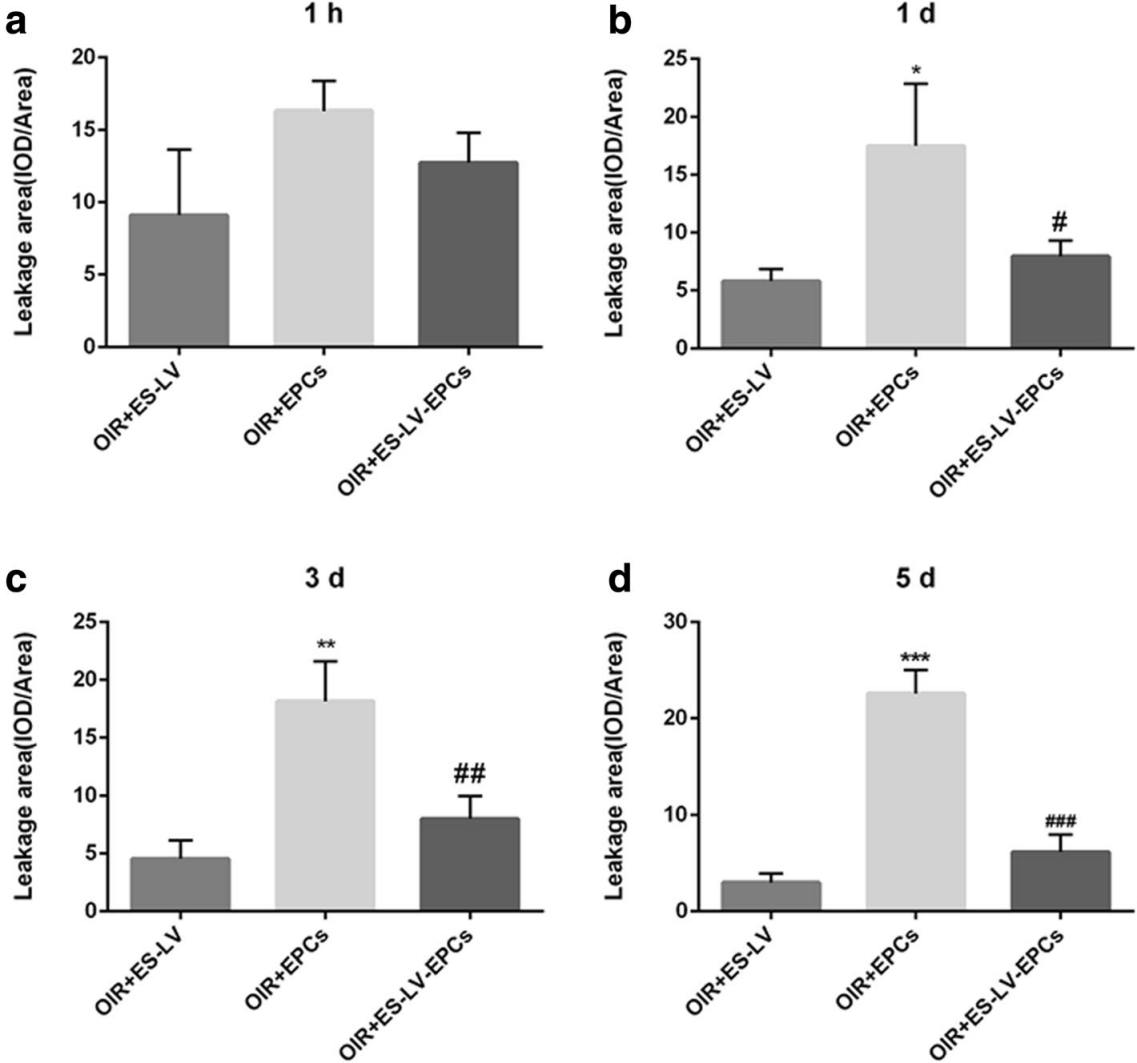
Neovascularization leakage areas analysis (IOD/Area, comparison in 3 groups). **a** Neovascularization leakage areas analysis at 1 h. **b** Neovascularization leakage areas analysis at 1d. **c** Neovascularization leakage areas analysis at 3d. **d** Neovascularization leakage areas analysis at 5d. ^*^P < 0.05, ^**^P < 0.01, ^***^P < 0.0001 as compared with OIR + endostatin-lentivirus; ^##^*P* < 0.01, ^###^*P* < 0.001 as compared with OIR + EPCs. OIR + ES-LV: OIR rats with endostatin-lentivirus intravitreal injection; OIR + EPCs: OIR rats with EPCs intravitreal injection; OIR + ES-LV-EPCs: OIR rats with endostatin-lentivirus-EPCs intravitreal injection

The neovascularization leakage area significantly reduced in the OIR + endostatin-lentivirus and OIR+ endostatin-lentivirus-EPC groups on day 5 (*P* < 0.001; Fig. 4). The size of this leakage area and the inhibiting effects on retinal neovascularization were similar between the two groups (Fig. 7), so it can be inferred that retinal neovascularization significantly reduced in both groups.

#### HE results

The nuclei in the vascular endothelium significantly reduced and were similar between the OIR+ endostatin-lentivirus and OIR+ endostatin-lentivirus-EPC groups (*P* < 0.05; Fig. 6a, b). The results of the HE method complied with those of fundus fluorescein angiography. It can be surmised that the inhibiting effects on retinal neovascularization were similar between endostatin-lentivirus and endostatin-lentivirus-EPC groups.

### Immunohistochemistry (IHC) results of endostatin, VEGF, and CD31 expression

**Fig. 8:**
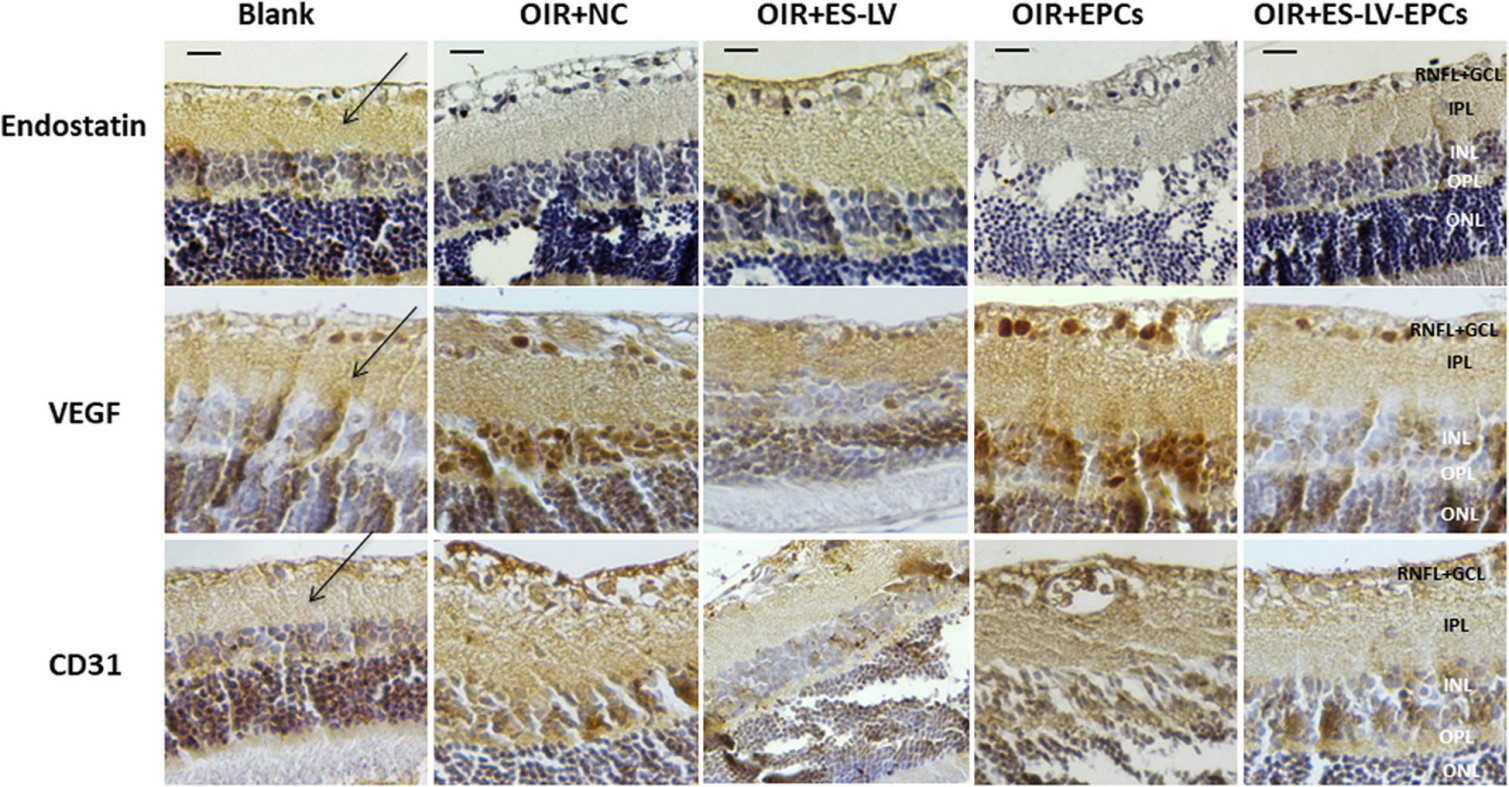
Photomicrographs of retinal sections labeled with primary antibodies against endostatin, VEGF, and CD31 (immunohistochemistry; magnification × 200). The positive staining is brown (indicated by a black arrow). RNFL+GCL (retinal nerve fiber layer and ganglion cell layer, black words); IPL (inner plexiform layer, black words); INL (inner nuclear layer, white words); OPL (outer plexiform layer, white words); ONL (outer nuclear layer, white words). Scale bar =25 μm. Blank: blank control group (age-matched rats kept in normoxia with non-intravitreal injection); OIR + NC (negative control group): Oxygen-induced retinopathy (OIR) rats with empty-lentivirus intravitreal injection; OIR + ES-LV: OIR rats with endostatin-lentivirus intravitreal injection; OIR + EPCs: OIR rats with EPCs intravitreal injection; OIR + ES-LV-EPCs: OIR rats with endostatin-lentivirus-EPCs intravitreal injection

**Fig. 9:**
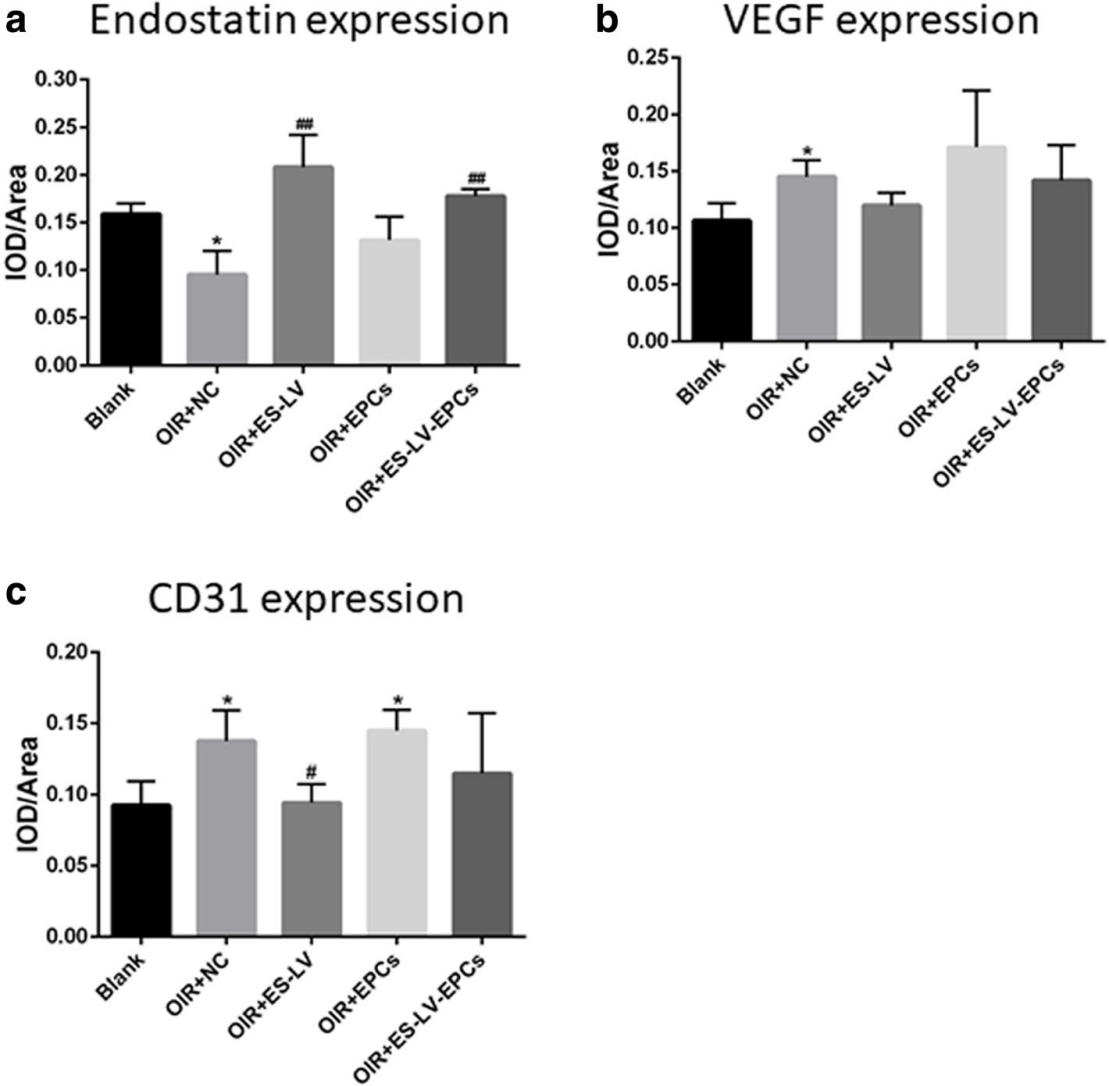
Endostatin, VEGF, and CD31 expression (IOD/Area, comparison in 5 groups). **a** Comparison of endostatin expression in the retinas of rats: ^*^P < 0.05 as compared with Blank; ^#^*P* < 0.05, ^##^*P* < 0.01 as compared with OIR + NC. **b** Comparison of VEGF expression in the retinas of rats: ^*^P < 0.05 as compared with Blank. **c** Comparison of CD31 expression in the retinas of rats: ^*^P < 0.05 as compared with Blank; ^#^*P* < 0.05 as compared with OIR + NC. IOD: integral optical density. Blank: blank control group (age-matched rats kept in normoxia with non-intravitreal injection); OIR + NC (negative control group): Oxygen-induced retinopathy (OIR) rats with empty-lentivirus intravitreal injection; OIR + ES-LV: OIR rats with endostatin-lentivirus intravitreal injection; OIR + EPCs: OIR rats with EPCs intravitreal injection; OIR + ES-LV-EPCs: OIR rats with endostatin-lentivirus-EPCs intravitreal injection

**Fig. 10:**
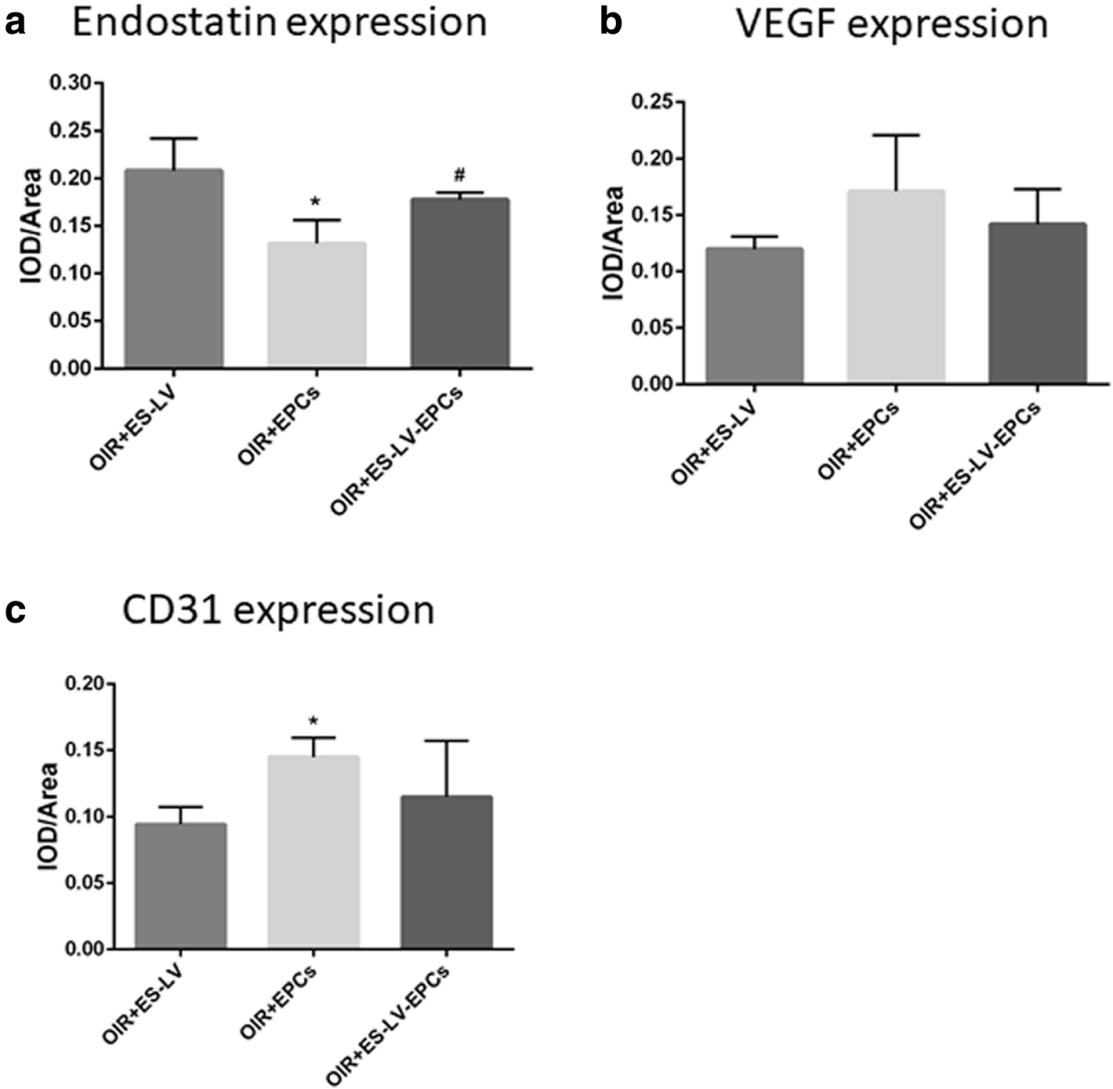
Endostatin, VEGF, and CD31 expression (IOD/Area, comparison in 3 groups). **a** Comparison of endostatin expression in the retinas of rats: ^*^P < 0.05 as compared with OIR + ES-LV; ^#^*P* < 0.05 as compared with OIR + EPCs. **b** Comparison of VEGF expression in the retinas of rats. **c** Comparison of CD31 expression in the retinas of rats: ^*^P < 0.05 as compared with OIR + ES-LV. IOD: integral optical density. OIR + ES-LV: OIR rats with endostatin-lentivirus intravitreal injection; OIR + EPCs: OIR rats with EPCs intravitreal injection; OIR + ES-LV-EPCs: OIR rats with endostatin-lentivirus-EPCs intravitreal injection

IHC revealed that the expression of endostatin presented mainly on the retinal nerve fiber layer (RNFL), ganglion cell layer (GCL) and inner plexiform layer (IPL); the positive staining of this method is brown color (Fig. 8). As compared with the blank control group, the level of endostatin expression decreased significantly in the OIR + NC group (*P* < 0.05); as compared with the OIR + NC group, the endostatin expression level increased significantly in the OIR+ endostatin-lentivirus and OIR+ endostatin-lentivirus-EPC groups (*P* < 0.01; Fig. 9), but there were no significant differences between the OIR+ endostatin-lentivirus and OIR+ endostatin-lentivirus-EPC groups (Fig. 10). The results showed that endostatin was overexpressed in the OIR+ endostatin-lentivirus and OIR+ endostatin-lentivirus-EPC groups, so it seemed that the overexpression of endostatin promoted the inhibition of retinal neovascularization. The results accorded with the fundus fluorescein angiography and HE results that retinal neovascularization was inhibited in these two groups.

IHC results (Fig. 8) showed that the VEGF expression existed mainly on RNFL, GCL, IPL, the inner nuclear layer (INL), outer plexiform layer (OPL) and the outer nuclear layer (ONL). Compared with the blank control group, VEGF expression significantly increased in the OIR + NC group (*P* < 0.05), but no significant differences were seen among the OIR+ endostatin-lentivirus, OIR + EPC, or OIR+ endostatin-lentivirus-EPC groups (Fig. 9). As compared with the OIR+ endostatin-lentivirus group, no differences were seen in the OIR + EPC or OIR+ endostatin-lentivirus-EPC groups (Fig. 10). Based on the results, VEGF expression did not increase in the OIR+ endostatin-lentivirus, OIR + EPC or OIR+ endostatin-lentivirus-EPC groups, so it appears that endostatin overexpression in the OIR+ endostatin-lentivirus and OIR+ endostatin-lentivirus-EPC groups may have decreased the VEGF expression. At the same time, the simple EPCs may have played a role in repairing the unhealthy neovascular tissues, which would explain why VEGF expression did not increase in the OIR + EPC group.

According to the IHC results, CD31 expression existed mainly on the RNFL, GCL, IPL, as well as the INL, OPL and ONL, which accorded with the VEGF expression findings (Fig. 8). Compared with the blank control group, CD31 expression significantly increased in the OIR + NC and OIR + EPC groups (*P* < 0.05), and no significant differences were seen in the OIR+ endostatin-lentivirus and OIR+ endostatin-lentivirus-EPC groups. However, CD31 expression decreased significantly in the OIR+ endostatin-lentivirus group (*P* < 0.05; Fig. 9), as compared with the OIR + NC group. Compared with the OIR+ endostatin-lentivirus group, no significant differences were observed in the OIR+ endostatin-lentivirus-EPC group (Fig. 10). CD31 expression did not increase in the OIR+ endostatin-lentivirus or OIR+ endostatin-lentivirus-EPC groups, which was similar to the findings for VEGF expression. These IHC results complied with the fundus fluorescein angiography and HE results, and CD31 served as a biomarker of ECs (as a part of neovascularization), it can be inferred that retinal neovascularization was inhibited in the OIR+ endostatin-lentivirus and OIR+ endostatin-lentivirus-EPC groups.

## Discussion

In the retina, VEGF is essential for angiogenesis, promotes retinal vascular permeability (breaking down the blood-retinal barrier), and participates in the pathological process of neovascularization in hypoxic conditions [27]. Moreover, VEGF may lead to the recruitment, migration, adhesion, proliferation, and organization of ECs and the formation of new vessels. VEGF can be secreted by several types of ocular cells [27, 28]. The role of VEGF in ocular neovascularization is critical, so VEGF is an attractive target for the development of gene therapies [8–11, 29, 30]. Therefore, new effective therapeutic tools that specifically target retinal neovascularization cells and decrease VEGF secretion are urgently needed. The increased expression of anti-VEGF molecules in active periods of neovascularization should reduce the potential for complications associated with a prolonged reduction in VEGF [30].

VEGF and endostatin represent a common component of inducers and inhibitors, respectively, in the process of angiogenesis [31]. Diabetic patients with low endostatin levels and high VEGF levels in the vitreous humor have a significantly higher risk of proliferative diabetic retinopathy progression after vitreous surgery than do those with high endostatin levels and low VEGF levels [32]. Therefore, a high endostatin level is beneficial for neovascularization therapy and is associated with a lower risk of neovascularization recurrence. For example, a previous study tested the hypothesis that an adenovirus vector (AVV) expressing endostatin would be as effective in reducing neovascularization in an OIR model as gene therapy with constitutively expressed endostatin [33]. However, the disadvantage of this in vivo study was that the virus safety was uncertain, so the effectiveness of the endostatin gene transfection strategy is questionable [14].

In the present study, a new gene therapy agent, endostatin-lentivirus-EPC, has been proven to inhibit retinal neovascularization and decrease VEGF expression significantly. Retinal neovascularization leakage was significantly reduced in the OIR+ endostatin-lentivirus-EPC group, and the nuclei in the vascular endothelium decreased significantly in the OIR+ endostatin-lentivirus-EPC group. Endostatin-lentivirus-EPCs have the following advantages:

1. Because EPCs can target neovascularization sites and the target gene can be secreted at the lesion site in neovascularization, the efficiency of the treatment is improved.
2. During the process of neovascular formation, a large number of EPCs are mobilized, migrated from bone marrow, and colonized in the sites of retinal ischemia; then, pathological neovascularization is initiated [21, 22]. Although the amount of EPCs increases in this process, the biological function of the EPCs is not improved, so the EPCs cannot repair the vascular endothelium; more importantly, the mobilized EPCs may promote the formation of new blood vessels [23]. Therefore, the transplantation of endostatin-lentivirus-EPCs into retinal tissue provides a sufficient number of healthy EPCs.
3. The endostatin secretion of endostatin-lentivirus-EPCs inhibits EC migration and decreases VEGF expression in the retinal tissue. The high VEGF level in the neovascular region is fundamentally solved through this strategy. Thus, retinal neovascularization leakage can be alleviated, and retinal neovascularization can be decreased.
4. It can be inferred from the fundus fluorescein angiography results that the adverse side effects associated with multiple intravitreal injections may be solved by a one-time endostatin-lentivirus-EPCs injection. Compared with the NC group, the use of endostatin-lentivirus injections in the OIR + endostatin-lentivirus group significantly inhibited neovascularization on day 3, but there was no significant difference until day 5 in the OIR + endostatin-lentivirus-EPC group, indicating that endostatin-lentivirus-EPCs had a later effect on neovascularization inhibition. If the genetically modified EPCs had a long-term effect on gene secretion, the temporary effect of direct injection of proteins and the side effects of repeated intravitreal injection could be solved.

The advantages of intravitreal injection are listed below. Firstly, the eyes are superficial and easy to operate. Secondly, the refractive medium of the eye is transparent, so EPCs could be injected into the target tissue through an intravitreal or subretinal injection under direct vision to reach the target retinal tissue. A transparent refractive medium of the eye provides an excellent operating basis for the intraocular transplantation of EPCs. The clinical effects of retinal neovascularization therapy could be observed intuitively through fundus fluorescein angiography or in fundus photographs. This therapy has good experimental operability and practicability. Finally, the vitreous cavity is less likely to cause an immune response if it is stimulated by external transplantation tissues or other antigens [34].

In this study, intravitreal injection of EPCs did not increase neovascular formation; these results are consistent with the results of a previous study [19]. Moreover, VEGF expression did not increase in the OIR + EPC group. The simple EPCs might have expedited the repair of the blood-retinal barrier [21], so retinal vascular permeability was alleviated simultaneously, and VEGF and CD31 expression did not increase. However, the observation time was too short, as human retinal neovascularization always occurs over a long period, this is a limitation of animal studies, as animals differ from humans.

## Conclusions

In conclusion, Endostatin-lentivirus-EPC is a new gene therapy agent that provides a novel therapeutic approach, and the present study only validates the feasibility of a preliminary idea. Therefore, in future studies, the amplified effects of transfected EPCs will be explored in vivo. The innovation of this study is the gene therapy targeting retinal neovascularization using EPCs as cellular vehicles, which provides a number of advantages for neovascularization therapy. It may also provide a basis for a new therapeutic direction for retinal neovascularization treatments.

## Methods

### Ethics

All procedures were performed in accordance with the Association for Research in Vision and Ophthalmology (ARVO) Statement for the Use of Animals in Ophthalmic and Vision Research. In addition, the Institutional Animal Care and Ethics Committee of Zhejiang University (Hangzhou, China) approved all animal experiments. All manipulations were performed following the rules outlined in our previous paper [35].

### EPC culture

Prior to the experimental procedures, Sprague-Dawley (SD) rats weighing 200–300 g (2–3 months old) were anesthetized with an intraperitoneal injection of sodium pentobarbital (30 mg/kg body weight; Merck KGaA, Darmstadt, Germany). EPCs were isolated, cultured, and identified as described previously [35]. Briefly, blood samples were obtained from the SD rats. After centrifugation in Ficoll-Paque Plus (GE Healthcare Bio-Sciences, Pittsburgh, PA, USA), the peripheral blood mononuclear cells were isolated; the cells were re-suspended in endothelial growth medium (EGM-2-MV; Lonza, Basel, Switzerland) and then plated into 6-well culture plates coated with fibronectin. The cultured medium was replaced every 2 d and non-adherent cells were removed thereafter.

### Recombinant lentivirus construction and transfection of EPC

The endostatin fragments were cloned by PCR. The recombinant lentiviral vector (endostatin-lentivirus-GFP) was produced by co-transfection of 293 T human embryonic kidney cells using Lipofectamine® 2000 reagent (Invitrogen, Grand Island, NY, USA). The primary EPCs were transferred into 6-well plates at 10^6^ cells/well for lentiviral transduction. The medium containing the recombinant lentiviral vector (endostatin-lentivirus-GFP) and Polybrene (5 μg/ml; Sigma Aldrich Corp.; St. Louis, MO, USA) was added at a multiplicity of infection of 100 to improve infection efficiency and was mixed with the cells. After incubation for 24 h, the cell-culture medium was removed and replaced with Dulbecco’s minimum essential medium supplemented with 10% fetal bovine serum. Cells were cultured for 96 h. Then, the relative expression levels of the endostatin gene were quantified by qRT-PCR. Non-transduced cells were used as the blank control, and cells transduced with GFP alone were used as the NC group.

For the qRT-PCR analysis, total RNA was extracted from the transfected EPCs using TRIzol**®** reagent (Invitrogen, USA). The relative expression levels of endostatin genes were quantified as previously described [35]. Briefly, using the 2^-ΔΔCt^ method, each sample was subjected to triplicate experiments: PCR products were incubated at 95 °C (3 min) and then run for 40 cycles at 95 °C (12 s) and 62 °C (40 s). The following primers were used: endostatin, forward: 5′-TCTCCCAAGTCGAAGACCCT-3′ and reverse: 5′-GAACAGCAGCGAAAAGTCCC-3′; GAPDH, forward: 5′-TCTCTGCTCCTCCCTGTTCT-3′ and reverse: 5′- ATCCGTTCACACCGACCTTC-3′. Results were normalized against GAPDH as a housekeeping gene control.

### Groups and animal treatment

On postnatal day (P) 7, the SD rats were randomly divided into experimental and control groups. According to some reports and our preliminary experiments [36–39], litters in the experimental group were exposed to hyperoxia (70% O_2_) and were fed by their mothers for 5 days (P7-P12) in a chamber and then returned to normal room air (20% O_2_) to induce oxygen-induced retinopathy (OIR). Oxygen concentration and room temperature were monitored and recorded three times per day. The control group was kept in normoxia (room air) under a normal diet and a 12 h light and dark cycle. Following euthanasia with pentobarbital sodium (0.5 mg/10 g body weight; Merck KGaA, Darmstadt, Germany) at different time points (P14, P15, P17, and P19), fundus fluorescein angiography (FFA) was performed for the experimental and control groups to confirm the established OIR rat model.

The age-matched rats under the normoxia condition with non-intravitreal injections were considered the blank control group. The OIR rats were used to compare the efficacy of intravitreal injections of endostatin-lentivirus, endostatin-lentivirus-EPC and simple EPC groups. The rats were randomized into five groups, as follows:

- Group I: Blank control group (normoxia and non-injection, *n* = 10).
- Group II: OIR + NC group (empty-lentivirus injection, 1 μg/μL, 1.5 μL, n = 10).
- Group III: OIR + endostatin-lentivirus group (endostatin-lentivirus injection, 1 μg/μL, 1.5 μL, n = 10).
- Group IV: OIR + EPC group (EPCs injection, 5 × 10^6^/mL, 1.5 μL, n = 10).
- Group V: OIR + endostatin-lentivirus-EPC group (endostatin-lentivirus-EPCs injection, 5 × 10^6^/mL, 1.5 μL, n = 10).

**Fig. 11:**
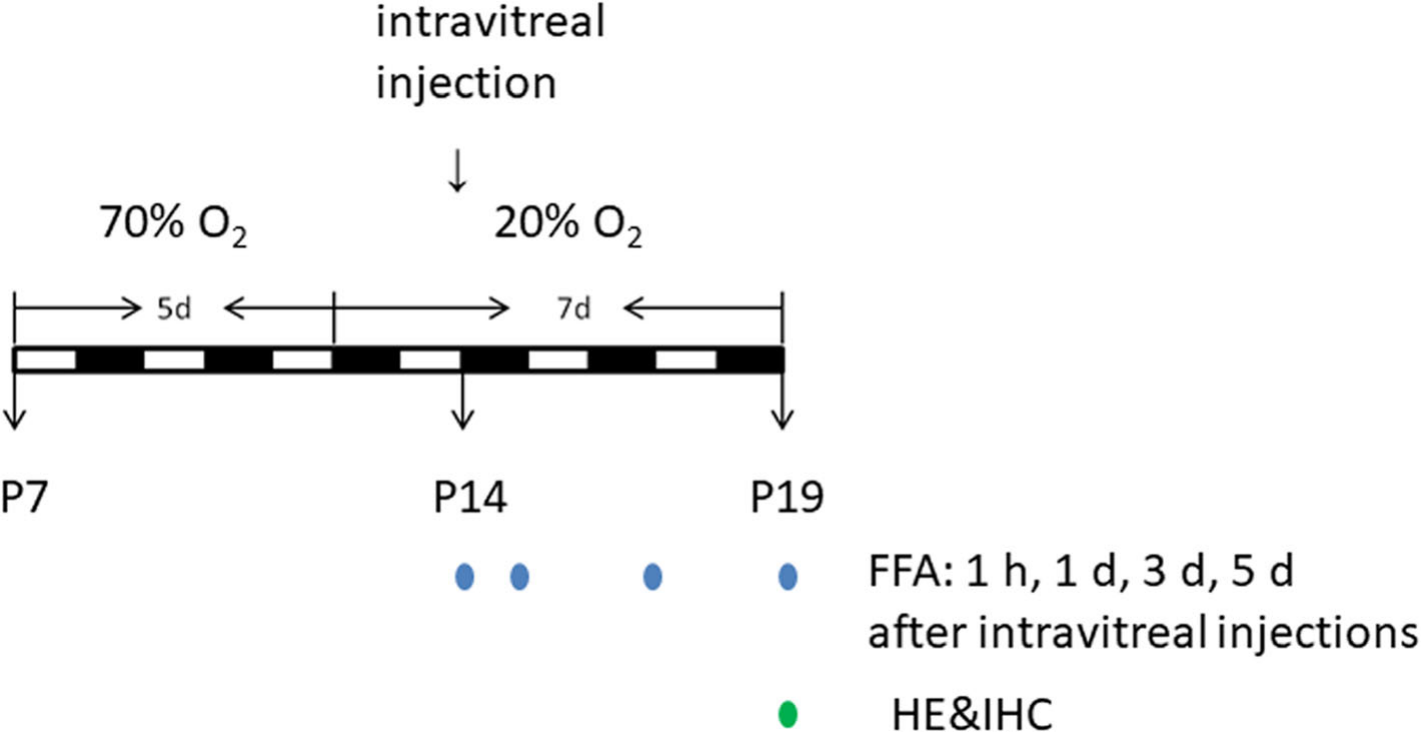
Detailed flow chart of animal treatment. FFA: fundus fluorescein angiography; HE: hematoxylin-eosin staining; IHC: immunohistochemistry; P: postnatal day

On P14, the right eye of each anesthetized rat received the same volume (1.5 μL) of intravitreal injection using a Hamilton syringe (#87900, Bonaduz, Switzerland) through the pars plana under a dissecting microscope, the injection concentration (5 × 10^6^/mL) was based on some reports and our preliminary experiments [40–42]; after injection, the animals were kept in normoxia until they were analyzed. Then, neovascularization leakage areas were compared using fundus fluorescein angiography at 1 h, 1 d, 3 d, and 5 d after intravitreal injections. Pups were sacrificed with an intraperitoneal overdose injection of pentobarbital on P19. Then the eyes were rapidly collected for histology analysis (HE and IHC). The Hematoxylin-eosin (HE) staining method was used to observe and count the number of nuclear cells in the ECs outside the retinal inner limiting membrane of the area affected by retinal neovascularization. Immunohistochemistry (IHC) was used to measure the retinal expression of endostatin, vascular endothelial growth factor (VEGF), and CD31. Figure 11 presents a detailed flow chart of animal treatment.

#### Fundus fluorescein angiography

Fundus fluorescein angiography was performed at 1 h, 1 d, 3 d, and 5 d after the intravitreal injections. Prior to fundus fluorescein angiography treatment, neonatal SD rats were anesthetized with a pentobarbital injection, and pupils were dilated with tropicamide phenylephrine eye drops (Santen Pharmaceutical Co. Ltd., Japan). Fluorescein sodium (10%, 75 mg/kg) was injected into the rats’ tail veins, and fundus fluorescein angiography images were immediately captured by a Heidelberg fundus fluorescein angiography instrument (Spectralis HRA, Germany). Three neonatal SD rats of each group were performed by fundus fluorescein angiography. The retinal neovascularization leakage area was assessed by outlining the border of each lesion using Heidelberg software and analyzed statistically.

#### HE staining

Three neonatal SD rats of each group were sacrificed at P19 after the last intravitreal injection; one eye of each rat was enucleated and fixed in 4% paraformaldehyde at 4 °C overnight. The orientation of the corneal limbus was marked at 12 o’clock of the corneal limbus. Then, the eyes were dehydrated and embedded in paraffin. Five-micrometer serial sections of whole eyes were cut sagittally through the cornea parallel to the optic nerve (without cutting the optic nerve). Each section was 30 μm apart and was stained with hematoxylin and eosin. The images were obtained and analyzed under a light microscope (Olympus, Japan). Three randomly selected areas in the sample coverslips were examined at a high magnification view (× 200). The number of nuclear cells in the ECs outside the retinal inner limiting membrane of the retinal neovascularization was counted and calculated, and the average value of each magnification view was used for statistical analysis.

#### IHC staining

Retinal expression of endostatin, VEGF, and CD31 was evaluated by the IHC method. The IHC staining procedure was performed following the manufacturer’s instructions. Unless otherwise stated, all washes were conducted 3 times for 5 min in PBS at PH 7.4 and performed at room temperature, while incubations were at 37 °C. Eye sections which were obtained by three neonatal SD rats from each group were deparaffinized and dehydrated, and then the antigens were repaired using a heated citric acid repair liquid (P0083; Beyotime Institute of Biotechnology, Shanghai, China).

Endogenous peroxidase activity was blocked by incubating the sections in 3% H_2_O_2_ for 20 min; then, the sections were washed and placed in goat serum for 10–15 min to block non-specific labeling. The sections were incubated at 4 °C overnight with one of the following primary antibodies (50 μL each): polyclonal rabbit anti-ES (1:200; ab202973; Abcam, UK), polyclonal rabbit anti-VEGF (1:200; 19,003–1-AP; Proteintech, USA), or polyclonal rabbit anti-CD31 (1:200; AF6191; Affinity, USA).

Negative controls were incubated on slides without any primary antibodies. Slides were washed and incubated for 15 min with 50 μL biotin-conjugated secondary antibody (1:1000; ab6802; Abcam, UK). Subsequently, a tertiary layer of streptavidin peroxidase was applied according to the manufacturer’s instructions (SABC-POD kit, Boster Biological Technology, Pleasanton, CA).

Antigen–antibody complexes were detected by incubation with diaminobenzidine (P0203; Beyotime Institute of Biotechnology, Shanghai, China) at room temperature for 3–30 min. Slides were counterstained with hematoxylin (Beyotime Institute of Biotechnology, Shanghai, China) for 3 min. Finally, the sections were washed, dehydrated, embedded in paraffin, and photographed. Positive cells were brown-stained in the cytoplasm and nucleus of the ganglion cell layer or inner nuclear layer, but no brown-stained cells were negative.

For immunocytochemical analysis, sections were coded and counted in a blind fashion on a light microscope (Olympus, Japan). A total of six visual fields from randomly selected areas in the sample coverslips were examined. Scopes were chosen as the percentage of positive cells that colocalized with endostatin, VEGF, or CD31. IHC staining gray scale was analyzed using Image-ProPlus (IPP) software (version 6.0, Media Cybernetics Inc., Rockville, MD, USA) and expressed as integral optical density (IOD). The average value of the IOD area was used for statistical analysis.

### Statistical analysis

The used software was SPSS 19.0 (IBM Corp., Armonk, NY, USA). A one-way analysis of variance was used to analyze all data. Values of *P* < 0.05 were considered statistically significant differences.

## Data Availability

The data that support the findings of the current study are available from Jing Ai or the corresponding author upon reasonable request.

## Abbreviations

VEGF: vascular endothelial growth factor
EPCs: endothelial progenitor cells
qRT-PCR: quantitative reverse transcription-polymerase chain reaction
EC: endothelial cell
OIR: oxygen-induced retinopathy
GFP: green fluorescent protein
HE: hematoxylin-eosin
IHC: immunohistochemistry
RNFL: retinal nerve fiber layer
GCL: ganglion cell layer
IPL: inner plexiform layer
INL: inner nuclear layer
OPL: outer plexiform layer
ONL: outer nuclear layer
AVV: adenovirus vector
SD: Sprague-Dawley
EGM: endothelial growth medium. IOD: integral optical density.
